# Out of the shadows: Phosphatase PP2A promotes shade avoidance by dephosphorylating phytochrome‐interacting factor 7

**DOI:** 10.1111/tpj.71158

**Published:** 2026-10-16

**Authors:** Brian C. Mooney

In dense vegetation, plants fiercely compete with their neighbours for sunlight. Shade‐avoiding species like *Arabidopsis thaliana* respond to vegetative shade with a series of adaptations collectively known as shade‐avoidance syndrome (SAS). SAS includes the elongation of hypocotyls and stems alongside upward leaf movement and a decrease in leaf blade area. These responses enable plants to grow beyond their neighbour's shadow and regain access to light (Casal, 2013).

Leaves primarily absorb red (R) light while reflecting far‐red (FR) wavelengths. Plants detect vegetative shade by monitoring the ratio of R:FR light using the photoreceptor phytochrome B (phyB) (Reed et al., 1993). When red light is abundant, the active ‘Pfr’ conformation of phyB translocates to the nucleus where it promotes the phosphorylation and subsequent degradation of PHYTOCHROME‐INTERACTING FACTOR (PIF) transcription factors. In shade (when R:FR < 1), phyB is maintained in the cytosol in an inactive ‘Pr’ state, allowing PIFs to accumulate and drive the expression of genes that promote SAS.

PIF7 has emerged as the central regulator of the shade avoidance response (Li et al., 2012). Its regulatory activity is partly controlled by reversible phosphorylation. Under white light, both phosphorylated and unphosphorylated forms co‐exist. Phosphorylated PIF7 is restricted to the cytoplasm, while the unphosphorylated form accumulates in the nucleus. However, nuclear phyB sequesters PIF7 in condensates leading to the suppression of its DNA‐binding activity. In shade, PIF7 is largely dephosphorylated. Coupled with the exclusion of inactive phyB from the nucleus, this enables PIF7 to activate the transcription of auxin biosynthesis and signalling genes, thereby promoting SAS‐related elongation. Despite the importance of this PIF7 phosphorylation switch, the enzymes responsible for its regulation have remained unknown.

Professor Enamul Huq at The University of Texas at Austin investigates how plants perceive and respond to light. Considering his group's long‐standing interest in phytochromes and PIFs, elucidating the regulation of PIF7 was a natural progression. In their new study, Huq's group revealed that phosphatase PP2A dephosphorylates PIF7 to promote shade avoidance (Cai et al., 2026). This work also included a collaboration with researchers specialising in phosphatase biology at Hebei Normal University, the previous home of first author Xingbo Cai.

PP2A is a heterotrimeric phosphatase comprising scaffold (A), regulatory (B) and catalytic (C) subunits. The Arabidopsis genome encodes 17 B subunit isoforms which are responsible for substrate specificity and subcellular localisation. Huq's group recently identified four PP2A B subunits that dephosphorylate another PIF family member, PIF3 (Cai et al., 2024). To determine whether these subunits also regulate PIF7 during shade avoidance, Cai et al. (2026) first examined hypocotyl elongation in a range of *Arabidopsis* PP2A mutants and overexpression lines. Double mutants *pp2ab"α‐pp2ab"β* (hereafter *b“αβ*) and *pp2ab′α‐pp2ab′β* (*b′αβ*) and the *pp2ab"αβ/ b′αβ* quadruple mutant (*4 M*) developed significantly shorter hypocotyls than wild‐type plants under shade, whereas overexpression of PP2A B subunits increased shade‐induced elongation. These results identified PP2A as a positive regulator of shade avoidance.

The authors next asked whether PP2A might regulate shade responses through direct interaction with PIF7. Yeast two‐hybrid experiments revealed that PP2A subunits B′α, B′β, B″α and B″β all interact with PIF7, and these interactions were subsequently confirmed *in vivo* by co‐immunoprecipitation. Genetic evidence further indicated that PP2A and PIF7 act in the same pathway. While the *4 M* mutant exhibited reduced hypocotyl elongation under shade, crosses with two independent *pif7* mutants produced plants resembling the *pif7* single mutant parent (Figure 1A,B). Conversely, the enhanced shade‐induced elongation caused by B*′*α overexpression was abolished in the *pif7* background (Figure 1C,D). These results indicate that PP2A acts upstream of PIF7 to promote shade‐induced growth.

**Figure 1:**
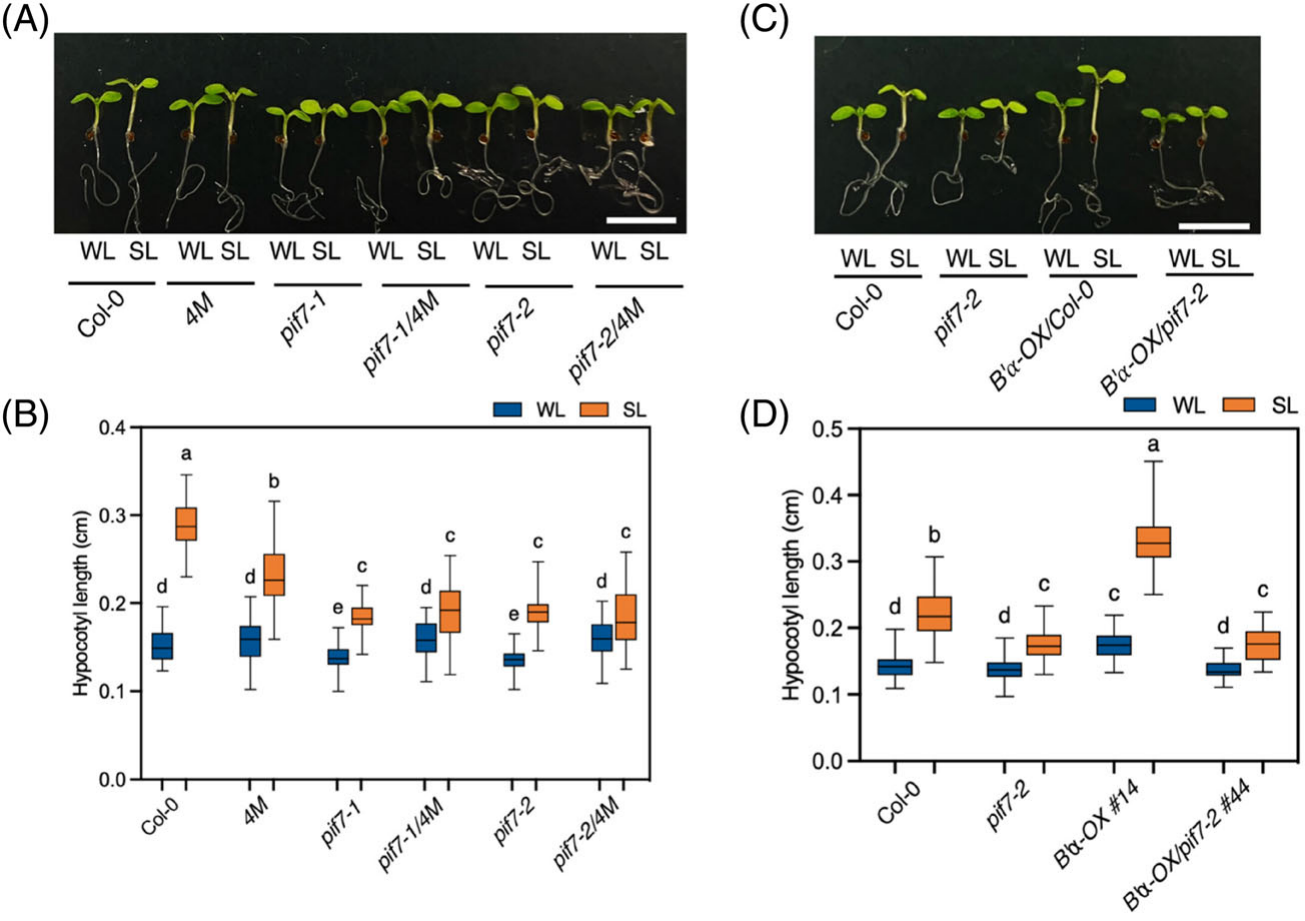
PP2A and PIF7 function in the same genetic pathway to regulate shade responses. (A, C) Arabidopsis seedlings grown under white light (WL) or shade light (SL). (A) PP2A B subunit quadruple mutant *4 M* and PIF7 mutants *pif7‐1* and *pif7‐2* show reduced hypocotyl elongation under shade compared to Col‐0 wild‐type seedlings. The quintuple *pif7‐1/4 M* and *pif7‐2/4 M* mutants resemble the *pif7* parental lines, indicating that PP2A acts upstream of PIF7 to promote shade avoidance. (C) PP2A B subunit overexpression line *B′α‐OX* shows an increased shade avoidance response which is lost in the *pif7‐2* background. (B, D) Boxplots showing quantification of hypocotyl lengths in A and C, respectively.

The authors then addressed the critical mechanistic question: does PP2A control the phosphorylation status of PIF7? Treatment with the phosphatase inhibitor cantharidin increased the abundance of phosphorylated PIF7 and delayed its rapid dephosphorylation following a transfer to shade. Similarly, phosphorylated PIF7 accumulated to higher levels in *pp2a* mutant *b"αβ* plants than in the wild type under both light and shade conditions. Most compellingly, *in vitro* assays confirmed that PP2A complexes efficiently dephosphorylate PIF7, providing direct biochemical evidence that PIF7 is a substrate of the PP2A phosphatase.

If PP2A‐mediated dephosphorylation activates PIF7, loss of PP2A should also compromise the transcriptional response downstream of PIF7. Indeed, shade treatment strongly induced the PIF7 targets *YUCCA8*, *YUCCA9*, *IAA19* and *IAA29* in wild‐type seedlings, but this response was markedly attenuated in the *4 M* mutant. Overexpression of the PP2A B subunits had the opposite effect, leading to increased expression of these genes under shade. Shade had only a minor effect on the expression of the *PP2A* regulatory subunits themselves, suggesting that PP2A regulates the shade response primarily through post‐translational control of PIF7 rather than through shade‐induced changes in *PP2A* expression levels.

Together, these findings support a model in which reversible phosphorylation provides a molecular switch connecting the light environment to growth. As the R:FR light ratio falls under vegetative shade, phytochromes shift towards the inactive Pr state and PP2A promotes the accumulation of dephosphorylated, active PIF7 in the nucleus. PIF7 can then activate auxin‐related shade‐responsive genes and drive elongation growth. The discovery may also have implications beyond PIF7. Huq's group previously found that PP2A interacts with PIF1, PIF3, PIF4 and PIF5 in yeast, while biochemical evidence now establishes both PIF3 and PIF7 as PP2A substrates (Cai et al., 2024, 2026). This raises the possibility that PP2A acts as a broader regulator of the PIF family. Determining whether other PIFs are genuine PP2A substrates and how PP2A achieves specificity among them will be important questions for future research.

By identifying PP2A as a phosphatase that activates PIF7, Cai et al. (2026) fill an important gap in our understanding of how plants translate changes in their light environment into developmental responses. In the longer term, Huq hopes that understanding these mechanisms could help inform strategies to optimise plant architecture and crop performance under dense planting conditions. For plants competing for their place in the sun, dephosphorylation of PIF7 provides one route out of the shadows.
